# Analysis of histopathological aspects in acquired middle ear cholesteatoma

**DOI:** 10.1016/S1808-8694(15)30143-9

**Published:** 2015-10-18

**Authors:** Adriana Leal Alves, Celina Siqueira Barbosa Pereira, Fernando de Andrade Quintanilha Ribeiro, Jose Humberto Tavares Guerreiro Fregnani

**Affiliations:** a PhD student in Otorhinolaryngology at FCMSCSP, Professor and Instructor in the Department of Morphology at FCMSCSP; b PhD in Medicine, Otorhinolaryngologist at FCMSCSP, Assistant Professor in the Department of Morphology at FCMSCSP; c PhD in Medicine, Otorhinolaryngologist at UNIFESP - EPM, Adjunct Professor in the Department of Otorhinolaryngology at FCMSCSP; d PhD in Medicine, Oncologist at Fundação Antônio Prudente, Assistant Professor in the Department of Morphology at FCMSCSP; e Send correspondence to: Adriana Leal Alves - R. Dr. João Clímaco Pereira 46 04532-070 SP SP. FAPESP

**Keywords:** cholesteatoma, histopathology, middle ear

## Abstract

Middle ear cholesteatomas are characterized by the presence of stratified squamous epithelium in this cavity with highly invasive properties causing bone destruction and it may lead to complications. **Aim**: To study the histopathological features in acquired cholesteatomas of the middle ear, correlating this data with patient age. Study design:clinical and experimental cross-sectional study. **Material and Methods**: Samples were obtained from 50 patients submitted to otologic surgery for the removal of middle ear cholesteatomas from 2006 to 2007. Thirty four patients were adults and 16 were children. Samples were studied by histological analysis. **Results**: we found the presence of epithelial atrophy (78%), epithelial acanthosis (88%), hyperplasia of the basal layer (88%) and formation of epithelial cones (62%). There was a positive and significant correlation between histopathological variables (such as epithelial acanthosis, hyperplasia of the basal layer and formation of epithelial cones). Histopathological variables presented no statistical significant difference in both age groups (p>0,05). **Conclusion**: Cholesteatomas have hyperproliferating characteristics with epithelial acanthosis, hyperplasia of the basal layer and the presence of epithelial cones in the matrix.

## INTRODUCTION

Middle ear cholesteatomas are characterized by the presence of keratinized squamous stratified epithelium inside the cavity. The middle ear is usually lined with pseudostratified ciliated columnar epithelium around the Eustachian tube and simple squamous columnar epithelium in the remaining of the area^1^.

Cholesteatoma epithelium has the four layers usually observed in thin skin epidermis, namely basal, squamous, granulous, and stratum corneum, and is called cholesteatoma matrix. It is supported by the loose connective tissue lamina propria, and contains collagen and elastic fibers, fibroblasts, and inflammatory cells, and is called cholesteatoma perimatrix^1^.

Cholesteatomas are a common occurrence in our area, but are rare entities in other countries, as they amount to 0.1% to 0.5% of all middle ear diseases. The ratio of men and women with disease is 1.2: 1.0 with ages ranging from 3 to 70 years^2^.

Cholesteatomas have migratory and lytic characteristics, and may damage the chain of ossicles and the mastoid cell bone tissue, leading to intra and extracranial complications^3^, ^4^.

Cholesteatomas can be congenital or acquired. Congenital cholesteatomas are made up of remainders of embryonal epithelium located mainly in the skull bones of patients without history of ear disease and preserved tympanic membrane. Acquired cholesteatomas set in after birth and are preceded by damage to the lax portion of the tympanic membrane (primary cholesteatomas) or generally marginal perforation of the tensioned portion of the ear drum (secondary cholesteatomas)^5^.

Cholesteatomas may present various histological patterns such as atrophy, acanthosis, basal layer hyperplasia, and presence of epithelial cones^4^, ^6^, ^7^.

Atrophy can be defined as cholesteatoma matrix thinning. Acanthosis is characterized by the proliferation of cells in the squamous layer resulting in thickened epithelium. Basal layer hyperplasia is defined by increases in the number of cells in the basal layer of the matrix, which in some cases may produce invaginations into the perimatrix called epithelial cones. Inflammatory process is characterized by intense perimatrix permeation by lymphocytes, neutrophils, plasmacytes, and macrophages^4^.

The objective of this study is to describe and correlate histopathologic patterns such as atrophy, acanthosis, basal layer hyperplasia, presence of epithelial cones, and inflammatory processes in middle ear acquired cholesteatoma fragments. We also looked at the correlation between these histopathologic patterns and age.

## MATERIALS AND METHOD

This is an observational cross-sectional study.

Our study included 50 cholesteatoma patients submitted to ear surgery for having clinical and physical examination history consistent with cholesteatoma chronic otitis media between 2006 and 2007.

Patients included in the study had to comply with the following criterion:

- Middle ear cholesteatoma found during surgery. Patients were excluded from the study based on the following criterion:
- Absence of cholesteatoma epithelium in hematoxylin-eosin (HE) stained slides under the microscope.

The patients were subdivided into two groups according to age. One of them contained individuals under 16 years of age (children) and the other had individuals above the age of 16 (adults).

Specimens were fixated in 10% formaldehyde, embedded in paraffin, sliced in a rotational microtome to produce 3 micron slices, and stained with hematoxylin-eosin for histological examination. Slides with cholesteatoma fragments were analyzed under an Axioscope 40 (Carl Zeiss do Brasil®) optical microscope with a 10x eye piece and 10x and 40x lenses. The microscope was coupled to a Sony CDC-IRIS® camera connected to a computer equipped with an image acquisition card to allow picture digitization with software program Axiovision 3.1.

The following histopathologic patterns were observed: atrophy (matrix with thickness of up to 4 keratinocyte layers), acanthosis, basal layer hyperplasia, epithelial cone formation, and associated inflammatory process; the latter can only be assessed in cases where the perimatrix is visualized under optic microscopy. Histopathologic patterns were assessed qualitatively (absent or present) and semi-quantitatively and graded according to intensity in a scale from 0 to 3 where: 0 - absent, 1 - mild, 2 - moderate, 3 - severe.

The collected information was stored in a computer database and statistically analyzed using software program Statistical Package for Social Science - SPSS® (release 13.0) for Windows. Comparisons between qualitative variables were performed using Pearson”s chi-square test and Fisher”s exact test, depending on the values expected in contingency Tables. The correlations between quantitative variables were assessed using Spearman”s ratio. All tests were done in a bicaudal manner considering statistical significance at 5%.

Free informed consent terms were signed by all patients so their data could be used anonymously and surgical specimens could be collected. This study was approved by the Research Ethics Committee of our institution in 2006 under permit 157/06.

## RESULTS

Our 50 patients were aged between 6 and 68 years (mean 30.4 years, standard deviation ± 19.2 years and median 24.5 years).

Thirty-two percent of our patients were under 16 (mean 11.4 ± 2.8 years). Sixty-eight percent of the patients were 16 and older (mean 39.3 ± 17.1 years).

Fifty-four percent of the subjects included in our study were males and 46.0% were females. In the group of children, 62.5% were males and 37.5% females. In the adult group, 50.0% were males and 50.0% were females.

**Figure 1:**
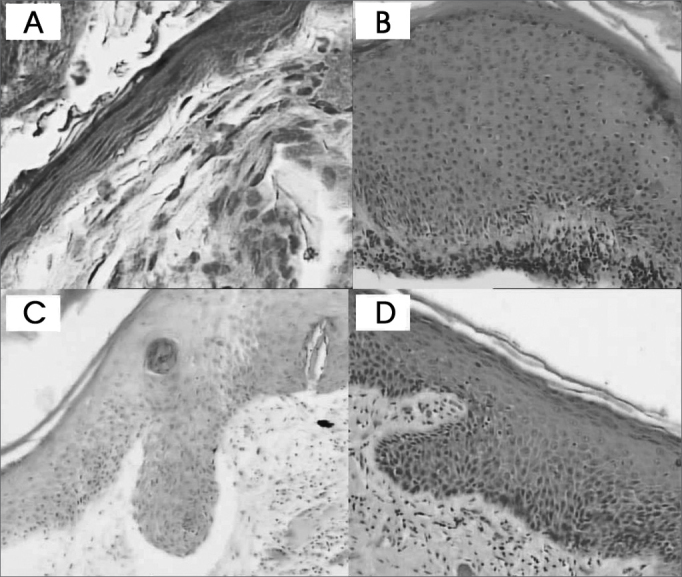
Cholesteatoma fragments: A- Atrophy (HE - 400x) B- Acanthosis (HE - 100x) C and D- Formation of epithelial cones (HE - 100x)

**Figure 2:**
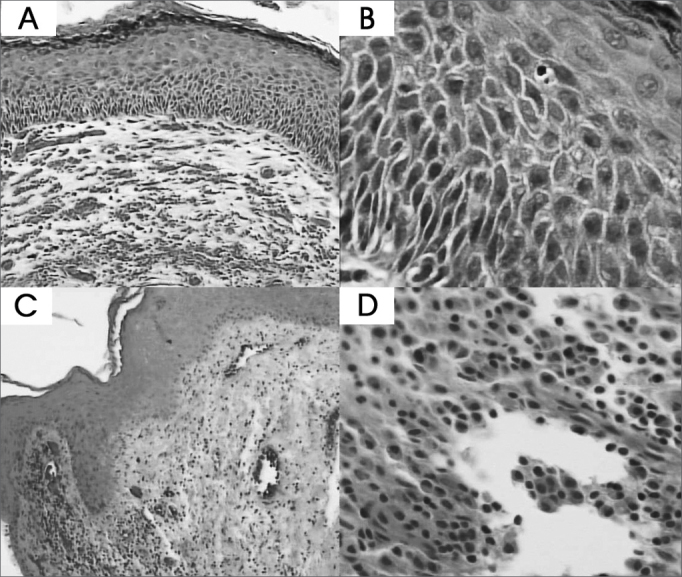
Cholesteatoma fragments: A and B- Basal layer hyperplasia (HE - 100x and 400x) C e D- Perimatrix inflammatory process (HE - 100x and 400x).

The cholesteatoma epithelium specimens analyzed were histologically heterogeneous, even in one same patient (Fig. 1 and 2).

In most cases, thinned squamous stratified epithelium areas (atrophy) were alternated with areas with normal thickness and overly thick epithelium. Fragments were categorized as presenting focal atrophy in 26 cases (52%), predominantly atrophic in 13 cases (26%), and atrophy-free in 11 cases (22%).

Eleven cases (22%) had severe acanthosis, 19 (38%) had moderate acanthosis, 14 (28%) had mild acanthosis, and 6 (12%) did not have acanthosis.

Five cases (10%) had severe basal layer hyperplasia, 19 (38%) had moderate basal layer hyperplasia, 23 (46%) had mild basal layer hyperplasia, and 3 (6%) had no hyperplasia.

Five cases (10%) had severe presence of epithelial cones, 13 (26%) had moderate presence of epithelial cones, 13 (26%) had mild presence of epithelial cones, and 19 (6%) did not have epithelial cones.

The inflammatory infiltrate found in the cholesteatoma perimatrix was predominantly lymphoplasmocytic of variable intensity with areas of neovascularization. As perimatrix was absent in three of the fifty fragments analyzed, we could not assess inflammation intensity in these cases. In ten (20%) of the remaining 47 cases it was severe, in 15 (30%) it was moderate, in 20 (40%) it was considered mild, and only in two (4%) was inflammation not present.

No histopathologic variable was significantly correlated with patient age (Table 1).

**Table 1:** Distribution of cholesteatoma samples according to age range and histopathologic pattern (atrophy, acanthosis, basal layer hyperplasia, presence of epithelial cones, and perimatrix inflammatory process).

		< 16 years		≥ 16 years		
VARIABLE	CATEGORY	n	(%)	n	(%)	P
	Absent	5	(31,3)	6	(17,6)	
Atrophy	Present	11	(68,7)	28	(82,4)	0,297(a)
	Absent	0	(0,0)	6	(17,6)	
Acanthosis	Present	16	(100,0)	28	(82,4)	0,159(a)
Basal layer hyperplasia	Absent	0	(0,0)	3	(8,8)	
	Present	16	(100,0)	31	(91,2)	0,542(a)
	Absent	6	(37,5)	13	(38,2)	
Epithelial cones	Present	10	(62,5)	21	(61,8)	0,960(b)
	Absent	1	(6,7)	1	(3,1)	
Inflammatory process	Present	14	(93,3)	31	(96,9)	0,541(a)

(a) Fisher”s exact test

(b) Chi-square association test

The correlations between histopathologic patterns are shown in Table 2.

**Table 2:** Correlation ratios of analyzed variables.

	Atrophy	Acanthosis	Basal layer hyperplasia	Cones	Inflammatory process
	rho	rho	Rho	rho	Rho
	(p)	(p)	(p)	(p)	(p)
Atrophy	___	-0,387 (0,006)	-0,390 (0,005)	-0,306 (0,031)	-0,182 (0,221)
Acanthosis	-0,387 (0,006)	___	0,722 (<0,001)	0,747 (<0,001)	0,387 (0,007)
Basal layer hyperplasia	-0,390 (0,005)	0,722 (<0,001)	___	0,627 (<0,001)	0,355 (0,014)
Cones	-0,306 (0,031)	0,747 (<0,001)	0,627 (<0,001)	___	0,556 (<0,001)
Inflammatory process	-0,164 (0,221)	0,387 (0,007)	0,355 (0,014)	0,556 (<0,001)	___

rho: correlation ratio

A negative significant correlation was found between the following variables: Ø atrophy and epithelial cones Ø atrophy and acanthosis Ø atrophy and basal layer hyperplasia

A positive significant correlation was found between the following variables: Ø acanthosis and epithelial cones Ø acanthosis and basal layer hyperplasia Ø acanthosis and inflammatory process Ø cones and basal layer hyperplasia Ø basal layer hyperplasia and inflammatory process Ø cones and inflammatory process

## DISCUSSION

This study included 50 cholesteatoma fragments. Other authors that analyzed a similar case base were Pereira^4^ and Dornelles^6^, with 31 and 74 cholesteatomas respectively.

The following histopathologic patterns were assessed: perimatrix inflammatory process, atrophy, acanthosis, basal layer hyperplasia, and formation of epithelial cones in the matrix. These patterns were assessed qualitatively (absent and present) and, when present, were analyzed semi-quantitatively and graded in mild, moderate, and severe depending on their intensity^4^, ^6^.

When looking at the hematoxylin-eosin stained specimens in an optical microscope, it was clear that cholesteatomas consist of keratinized squamous stratified epithelium, with four layers identical to those of thin skin (basal, squamous, granulous, and stratum corneum), lying on a bed of connective tissue that contains fibroblasts, lymphocytes, plasmacytes, and e macrophages. These findings were compatible with those of other authors that looked into cholesteatoma histopathology^1^, ^4^, ^6^. There is some divergence as to the best nomenclature for cholesteatoma epithelium and the connective tissue it lies on. Lim, Saunders^1^ called the epithelium matrix and the connective tissue perimatrix. This classification was adopted by a number of authors^3^, ^4^, ^6^, ^7^, ^8^, ^9^, ^10^. Few authors however prefer the terms epithelial and subepithelial tissue^11^, ^12^, ^13^. The classification initially suggested by Lim, Saunders^1^, that divides cholesteatomas into matrix and perimatrix, was adopted in this study.

In our study the perimatrix could only be analyzed in 47 (94.0%) of the 50 cases. Absent perimatrix was also observed by Pereira4 as only it could only be visualized in 22 (71.0%) of 31 cases.

A possible explanation for this event is that the perimatrix was too thin to be detected under optical microscopy. Lim, Saunders1 observed that all cholesteatomas had a perimatrix, but occasionally, depending on its thickness, it could only be identified with the aid of transmission electron microscopy. They believed that, in these cases, collagen fibers would be practically absent, indicating collagenase activity, an enzyme present in cholesteatomas that destroys collagen fibers. Another fact supporting this idea is the pressure applied by the content of the cholesteatoma keratin lamellae upon matrix and perimatrix, compressing these tissues against the bone framework of the middle ear. Both collagenase activity and the pressures applied on the bone walls of the middle ear are factors that may explain the bone erosion that accompanies cholesteatomas^1^, ^4^,. Collagenases are currently referred to as metalloproteinases and are present in middle ear cholesteatoma perimatrix^14^.

In our study the perimatrix was visible in 47 cases (94.0%). Inflammation was severe in 10 (21%), moderate in 15 (32%), mild in 20 (43%), and only in two cases (4%) was it absent. Therefore, 96% of the cases had some degree of inflammation. These results are similar to those described by Pereira^4^, who reported inflammation rates of 90.9%, and agree with clinical observation findings as scarcely ever are middle ear cholesteatomas not accompanied by an inflammatory process. The most frequently found inflammatory cells in the perimatrix are lymphocytes, plasmacytes, and macrophages, as also seen by other authors^4^, ^15^. The inflammatory process present in the cholesteatoma perimatrix seems to play an important role in inducing the production of cytokines^16^, ^17^. These proteins can modify the characteristics of the very cells that produced them and of adjacent cells^18^, ^19^. The cascade effect of cytokines and the interaction between the various types of cytokines end up being both the cause and the effect in the aggressive characteristics of cholesteatomas^20^.

Although we looked into inflammation, the main purpose of this study was to assess matrix-related aspects, and thus the cases in which the perimatrix could not be observed in the optic microscope were not excluded. This is different from Dornelles^6^, whose purpose was to describe perimatrix thickness. This author used inexistent perimatrix as a criterion for exclusion^6^.

The following distribution was observed for atrophy: 52% of the cases were categorized as focal atrophy, 26% as predominant atrophy, and 22% did not have atrophy. This finding is different from what was observed by Pereira^4^, as the author reported absent atrophy in only 16.1% of the cases. Pereira^4^ also observed an alternation between areas with atrophy and acanthosis, but categorized these variables in a different way (atrophy alone, atrophy predominance, balance between variables, acanthosis predominance, and acanthosis alone). Atrophy occurs as cells reduce in size and lose structural components such as endoplasmic reticulum and mitochondria. When a significant number of cells are involved, the tissue becomes atrophic. This process derives from a number of causes, such as reduced blood supply, inadequate nutrition, lack of use, compression, and aging. As apoptosis can be triggered by these same mechanisms, atrophy may progress up to the point when cells die. Some authors showed that apoptosis induction substances (p 53, bax) are present in high concentrations in acquired middle ear cholesteatomas^20^, ^21^. It is possible that the atrophic areas found in our study were those in which there was increased compression by cholesteatoma content or greater expression of apoptosis induction cytokines.

Acanthosis was absent in 6 (12%) of the 50 cases analyzed. This result is similar to reports by Pereira4, who indicated absent acanthosis in 16% of the cases. As acanthosis means increased epithelial thickness caused by larger numbers of cells in the squamous layer, one may infer that in middle ear cholesteatomas there is proliferation of cells in this layer. Dornelles6 also looked at matrix thickness using a different analytical method that involved counting matrix cells.

Atrophy and acanthosis were negatively and significantly related, showing that they are inversely proportional to each other. The more atrophic an area is, the less acanthosis will it have and vice-versa. This finding is in agreement with the literature^4^.

Basal layer hyperplasia was absent in 6 (12%) of the 50 studied cases. Epithelial cones were absent in 19 (38%) cases. Pereira^4^ also analyzed these variables but in a combined manner, and found that in 59% of the cases both were absent, differently from what we found in our study. The fact that cholesteatomas had basal layer hyperplasia and eventually formed epithelial cones indicates that this disease can be hyperproliferative, with increased number of cells in the basal layer forming invaginations into the perimatrix. Many authors^15^, ^22^ found increased inflammation around epithelial cones, but this finding was not observed by Pereira^4^. Our study used Spearman”s rank correlation test to show the associations between presence of inflammation, acanthosis, basal layer hyperplasia, and epithelial cone formation, showing that the greater the inflammatory process, the greater the chances of acanthosis, basal layer hyperplasia, and presence of epithelial cones in cholesteatoma fragments.

Other positive significant correlations were found between histopathologic patterns (acanthosis, basal layer hyperplasia, and presence of epithelial cones) showing greater proliferation of keratinocytes in cholesteatoma epithelium, as also reported by Pereira^4^.

None of the histopathologic patterns analyzed in the cholesteatoma matrix were significantly related to patient age group. This is different from the findings reported by Dornelles^7^ in which perimatrix thickness was larger in children than in adults. This author considered subjects aged up to 18 as children. In reality, the division into two age groups does not necessarily correspond to the existence of cholesteatomas in children or adults, but to cholesteatomas removed from patients when they were children or adults. One should consider the time of evolution of cholesteatomas, and not the age at which they were removed. Maybe that is why we failed to find statistically significant differences between the two age groups.

We may infer that cholesteatomas with more epithelial cones, basal layer hyperplasia, and acanthosis (patterns easily verified in conventional histopathologic tests) have hyperproliferative characteristics. Thus, surgical specimens of patients operated for cholesteatoma must be sent for pathology tests. Patients with such characteristics identified in pathology tests must be clinically followed more closely than individuals without increases in these variables.

## CONCLUSION

Acquired middle ear cholesteatoma is a hyperproliferative disease accompanied by acanthosis, basal layer hyperplasia, and formation of epithelial cones in the matrix and inflammatory processes in the perimatrix. The more acanthosis was found in the cholesteatoma matrix, the greater was basal layer hyperplasia. The more epithelial cones were found into the perimatrix, the more acanthosis and basal layer hyperplasia were present in the cholesteatoma matrix. Positive and statistically significant correlations were found between perimatrix inflammation and acanthosis, and basal layer hyperplasia and formation of cones in the cholesteatoma matrix.

There were no statistically significant differences in the histopathologic patterns observed in the cholesteatoma matrix of pediatric and adult patients.

## References

[1] Lim DJ, Saunders WH (1972). Acquired cholesteatoma: light and electron microscopic observations. Ann Otol Rhinol Laryngol.

[2] Gyo K, Sasaki S, Yasuyuki N (1996). Residue of middle ear cholesteatoma after intact wall tympanoplasty: surgical findings at one year. Ann Otol Rhinol Laryngol.

[3] Pereira CSB (1997). Análise de estudos da expressão das citoqueratinas no colesteatoma adquirido. Tese (Mestrado).

[4] Pereira CSB (2001). Imunoexpressão da citoqueratina 16 e do Ki-67 no colesteatoma adquirido. Tese (Doutorado).

[5] Shambaugh G, Shambaugh G. (1967). Surgery of the ear.

[6] Dornelles CD (2004). Colesteatomas adquiridos: análise comparativa da perimatriz entre pacientes pediátricos e adultos. Tese (Mestrado).

[7] Dornelles CD, Costa SS, Meurer L, Schweiger C (2005). Comparação da espessura da perimatriz, de colesteatomas adquiridos, entre pacientes pediátricos e adultos. Rev Bras Otorrinolaringol.

[8] Sudhoff H, Bujia J, Holly A, Kim C, Fisseler-Eckhoff A (1994). Functional characterization of middle ear mucosa residues in cholesteatoma samples. Am J Otol.

[9] Ottaviani F, Neglia CB, Berti E (1999). Cytokines and adhesion molecules in middle ear cholesteatoma. A role in epithelial growth?. Acta Otolaryngol (Stockh).

[10] Mallet Y, Nouwen J, Lecomte-Houcke M, Desaulty A (2003). Aggressiveness and quantification of epithelial proliferation of middle ear cholesteatoma by MIB-1. Laryngoscope.

[11] Schulz P, Bujia J, Holly A, Schilling V, Kastenbauer E (1993). Possible autocrine growth stimulation of cholesteatoma epithelium by transforming growth factor alpha. Am J Otolaryngol.

[12] Ergün S, Zheng X, Carlsöö B. (1996). Expression of transforming growth factor-alpha and epidermal growth factor receptor in middle ear cholesteatoma. Am J Otol.

[13] Chung JW, Yoon TH (1998). Different production of interleukin-1a, interleukin-1b and interleukin-8 from cholesteatomatous and normal epithelium. Acta Otolaryngol (Stockh).

[14] Morales DSR, Penido NO, Da Silva IDCG, Stávale JN, Guilherme A, Fukuda Y (2007). Matriz metaloproteinase 2: um importante marcador genético para colesteatomas. Rev Bras Otorrinolaringol.

[15] Schilling V, Bujía J, Negri B, Schulz P, Kastenbauer E. (1991). Immunologically activated cells in aural cholesteatoma. Am J Otolaryngol.

[16] Ribeiro FAQ, Pereira CSB (2002). Sociedade Brasileira de Otorrinolaringologia. Tratado de Otorrinolaringologia.

[17] Alves AL (2004). Análise da expressão das citocinas no colesteatoma adquirido da orelha média. Tese (Mestrado).

[18] Hamblin AS (1993). Cytokines and cytokine receptors.

[19] Abbas AK, Lichtman AH, Pober JS, Abbas AK, Lichtman AH, Pober JS (1998). Imunologia celular e molecular.

[20] Albino AP, Kimmelman CP, Parisier SC (1998). Cholesteatoma: a molecular and cellular puzzle. Am J Otol.

[21] Tomita S (2000). Aspectos moleculares do colesteatoma - imunoexpressão das proteínas controladoras do ciclo celular: p53, bax e bcl-2. Tese (Doutorado).

[22] Mayot D, Béné MC, Perrin C, Faure GC (1993). Restricted expression of Ki-67 in cholesteatoma epithelium. Arch Otolaryngol Head Neck Surg.

